# Intraspecific Interactions Decrease Stress Affecting Welfare in Shelter Dogs: A Comparison of Four Different Housing Conditions

**DOI:** 10.3390/ani13111828

**Published:** 2023-05-31

**Authors:** Sara Corsetti, Eugenia Natoli, Rupert Palme, Emanuela Viggiano

**Affiliations:** 1 School of Agriculture and Environment, The University of Western Australia, Crawley, WA 6009, Australia; 2 Canile Sovrazonale, ASL Roma 3 (Local Health Unit Rome 3), 00148 Rome, Italy; enatoli@tiscali.it; 3 Department of Biomedical Sciences, Vetmeduni, 1210 Vienna, Austria; rupert.palme@vetmeduni.ac.at; 4 Ministero della Pubblica Istruzione, 20100 Milano, Italy; emanuela.viggiano@yahoo.it

**Keywords:** domestic dog, shelter, welfare, behaviour, cortisol

## Abstract

**Simple Summary:**

Shelters are stressful environments for domestic dogs (*Canis familiaris*). Evaluating dogs’ welfare is crucial to improve their life condition and to promote a better management of shelters. The aim of this research was to analyse the physiological and behavioural responses of dogs in different environmental conditions. We conducted behavioural observations on 10 male dogs and collected faecal samples in order to determine the level of cortisol metabolites. Dogs were observed in four different cage conditions: (i) alone in a cage; (ii) alone in an enriched cage; (iii) in cage with conspecifics; (iv) in cage with regular interaction with humans outside the cage. The presence of conspecifics is the best way to reduce stress in shelter dogs. This research could provide some useful guidelines for managing shelters and improving dogs’ life condition.

**Abstract:**

Shelters are stressful environments for domestic dogs (*Canis familiaris*). Evaluating dogs’ welfare is crucial to improve their life condition and to promote a better management of shelters. We aimed at verifying which variables improved welfare in 10 shelter dogs ((hosted in the shelter “Centro cinofilo Caerite” in Bracciano (Rome)) by analysing their behavioural responses in different environmental conditions. Furthermore, faecal samples were taken to measure cortisol metabolites (CM), a non-invasive method to evaluate adrenocortical activity in dogs. Dogs were observed for a total of 400 h in 4 different cage conditions: (i) alone in a cage; ii) alone in an enriched cage; (iii) in cage with conspecifics; (iv) in cage with regular interaction with humans outside the cage. Alone in the cage situation showed highest frequencies of displacement activities (Friedman test: χ^2^ = 13.32; *p* = 0.004). In contrast, being in the cage with conspecifics seems to reduce displacement activity frequency, as well as the level of faecal cortisol metabolites (Friedman test: χ^2^ = 8.04; *p* = 0.045). Our results suggest that conspecifics’ presence is the best way to reduce stress in shelter dogs. This research could provide some useful guidelines for managing shelters and improving dogs’ life condition.

## 1. Introduction

In Italy, where the no-kill policy for dogs (*Canis familiaris*) has been enforced by law since 1991, evaluating dogs’ welfare in shelters is crucial to minimize stress factors in order to improve dogs’ life condition. A more responsible management of shelters is highly desirable especially when dogs are kept in shelters for life, if judged unadoptable. Animal welfare is defined as the state of an individual in relation to its attempts to adapt to the environment [1]. Adapting means having control of physical and mental stability [1]. In other words, welfare concerns measurable individual characteristics and varies along an axis, whose extremes are very good or very poor. Poor life conditions influence animal behaviour and physiology and could cause pre-pathological or pathological conditions.

Shelters are stressful environments for dogs [2,3,4,5,6,7,8,9,10,11]. Dogs have to face, for example, spatial and social constriction, exposure to a novel environment, noise, eventual separation from an attachment figure [6,12]. All these variables have one thing in common: unpredictability. Any event that is new, unpredictable or unknown to the individual indicates that the current situation is not fully understood and may suggest probable imminent harm [13]. Due to these conditions, sheltered dogs seem to be more predisposed than pets to show stereotypic behaviours, hyperactivity, fearfulness, continual barking and/or behaviours that indicate anxiety, such as displacement activities [7,14,15,16,17] As defined in [14]: ”Displacing activities are behaviour patterns (mostly body care activities) characterized by their apparent irrelevance to the situation in which they appear. [...] Displacement activities tend to occur in situations of psycho-social stress”.

Stress activates the hypothalamic-pituitary-adrenal (HPA) axis [6,7,13], and it consequently provokes a hypersecretion of glucocorticoids [18], making individuals more vulnerable to stress disorders.

Environmental enrichment could be a way to reduce shelter dogs’ stress and improve their life condition. In shelter dogs, it has been demonstrated that animate and inanimate enrichments—characterized by human contact and food-filled toys, respectively—influence dogs’ behaviour and help to prevent undesirable behavioural patterns (reviewed in [13,19,20,21,22]).

Some studies underlined that the presence of conspecifics could reduce stress in shelter dogs (reviewed in [13]), but none compared which type of situation—enrichment, no enrichment, presence of conspecifics or interaction with humans—has a bigger influence on dogs’ behaviour and welfare.

The aim of this research was to study which variables contribute to better welfare in shelter dogs utilising one physiological indicator (faecal cortisol metabolites) and some behavioural ones in different environmental conditions. The measurement of faecal cortisol metabolites (FCMs) is a non-invasive method to evaluate adrenocortical activity in dogs [23,24,25]. Results could suggest some guidelines for shelter management to improve dogs’ life quality.

## 2. Materials and Methods

### 2.1. Animals and Housing

The animal sample consisted of 10 male dogs (between two and five years old) that are healthy and non-neutered. They entered the dog shelter “Centro cinofilo Caerite” in Bracciano (Rome) between one to two years before the study began. This private dog shelter also housed dogs caught free-ranging in the territory of some neighbouring municipalities. Dogs were housed in single cages of 4.5 m2 with an indoor and outdoor area. The dogs’ cages were cleaned with running water twice a day, while the dog was in another part of the cage.

Before the beginning of the study, dog’s health status was assessed through a clinical routine examination, i.e., temperature control, visual physical examination of the ears and mouth; checking and treatments for endo- and ectoparasites. In order to avoid further stress to the animals, the clinical examination was done just once upon intake; however, the dogs’ health was monitored through the study to spot any symptoms (e.g., diarrhoea, vomit and/or cough).

Due to management restrictions, dogs were never taken out the cage for a walk. An exception was made for the dogs involved in this research who were taken for walks outside the cage for ten consecutive days.

### 2.2. Behavioural Observations

The behavioural observations were conducted between December 2002 and November 2003 by a previously trained observer, who sat in front of the cage but was concealed by a cover. She never interacted with the dogs, who thus quickly became accustomed to her presence, perceived only through olfactory cues.

Individuals were observed in 4 different situations:

1. Alone in a cage (baseline condition): the dog was housed in a cage (dimensions 1.5 m × 3 m), delimited by a cement wall 1 m high and, above it, another meter of a narrow knitted wire mesh. The visibility of adjacent cages was very scarce, so dogs could hear and smell but not see each other. In this cage, there were only bowls for water and food.
2. Alone in an enriched cage: this cage (dimensions 3 m × 3 m), was delimited with a wide-meshed wire mesh, and so dogs could see conspecifics housed in adjacent cages. It was enriched with a dog basket, a platform heighted about 80 cm (from which they could easily see other dogs in other cages), toys, bones and clothes impregnated with odours. Furthermore, attached to the cage was a small space (3 m × 2 m) with earth and grass to give dogs the opportunity to dig and/or eat grass. To amplify the effect on dogs’ behaviour, we increased the quantity of the environmental enrichment.
3. In a cage with conspecifics: this cage had the same characteristics as the enriched cage but, instead of inanimate enrichment (which was not present), there were two neutered females.
4. Interactions with humans: the dog was alone in a cage (dimensions 1.5 m × 3 m), but with regular daily interactions (same time of the day) with the same person, outside the cage. This cage had the same features as the cage in situation number one but, on a daily basis, the dog was regularly led by shelter staff to an outdoor enclosure where it interacted with the person (play, cuddle, as the dog chose), and it could move freely. The behavioural observation was carried out exclusively by the observer when the dog was in its cage.

In each situation, dogs were observed one hour a day at different times of the day (to cover the entire daylight range) for 10 consecutive days, interspersed with a period of two days at each change of situation (the dog was moved to the new cage), for a total of 40 h/dog.

Behavioural data were collected with the “Focal Animal Sampling” method (one dog a time), using “All occurrences” (which records the number of times the dog exhibits a specific behaviour, e.g., the number of times it scratches itself) and “One/zero” (which records the number of predetermined intervals—in this case 60 s—in which the dog exhibits a behaviour, e.g., the number of intervals in which it barks) [26]. We utilised an ethogram previously described in [10]; the behavioural patterns utilised in the statistical analysis of this paper are reported in Table 1. To gather the behavioural patterns in categories, we utilized one of the conventional criterion in ethology, the consequential evidence (reviewed in [26]), which is based on grouping together behaviours that have the same function. These criteria have been used in previous studies (i.e., [9,10,11]) (Table 1).

It is known that in shelters, a dog that starts barking (or howling) almost always drags the other dogs into the barking (or howling) activity, especially if housed in the cages in the same corridor [27]. There is some indication that in the original environment of adaptation, collective barking and howling had the function of intimidating rival packs even at considerable distance in order to keep them away from resources [28].

In this study, during the collection of behavioural data, the observer distinguished between behaviours collected when the other dogs were quiet and behaviours collected when all dogs were barking together, in order to ascertain whether the dogs’ behaviour was influenced by the general emotional state expressed by the vocalisations.

### 2.3. Determination of Cortisol Metabolites in Faecal Samples

In order to assess the level of faecal cortisol metabolites (FCMs), at least 3 faecal samples in each situation were collected for each dog (see Table 2), for a total that ranged from 18 to 28 samples/dog. Faecal samples were collected in consecutive days if possible, but always within the ten days of behavioural observation. Faeces were collected only when excreted in the presence of the observer and when the dog was in another part of the cage. Samples were frozen within 1 h from emission.

FCMs were determined as described earlier in [23]. Briefly, 0.5 g of each well homogenized faecal sample were weighted and mixed with 5 mL 80% methanol. Following shaking (30 min) and centrifugation (2.500× *g*; 15 min), an aliquot of the supernatant (after a 1 + 9 dilution with assay buffer) was analysed (in duplicate, where analysis was repeated if CV% was higher than 10%.) in an in-house cortisol enzyme immunoassay, previously validated for use in dogs (details are given in [24,29]).

### 2.4. Statistical Analysis

All data were analysed with the Friedman test, which is the non-parametric alternative for a repeated-measures ANOVA, and Kendall’s concordance coefficient to related samples was calculated; the Bonferroni correction for multiple comparisons was applied. Data analysis was conducted using the IBM SPSS software.

## 3. Results

The behaviour of the dogs at silent times compared to when all the dogs barked together were correlated, with the exception of one (attention; Table 3). Based on these results, we decided to use the total number of occurrences of each behaviour for statistical analysis, without distinguishing between moments of silence and moments when all the dogs were barking.

**Figure 1:**
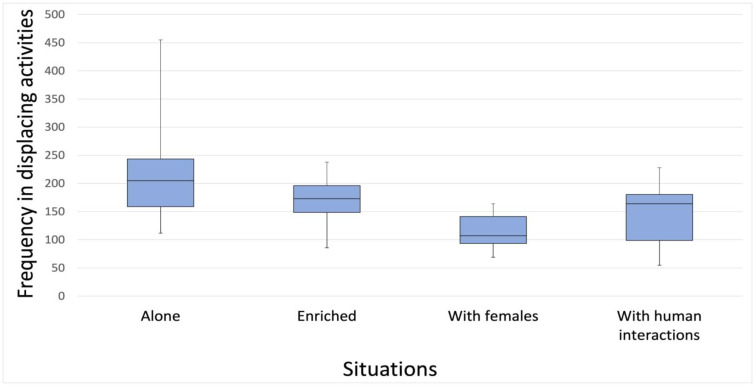
Average frequencies of displacement activities in the four different situations. The graph shows the frequencies of displacement activities in the different situations: alone in cage, alone in an enriched cage, in cage with two neutered females, and alone in cage with daily regular human contact ouside the cage. The black bars within the box plots indicate the median; the dots represent the outliers; and the whiskers represent the min and max value.

Displacement activities were more frequent when dogs were alone in the cage than when in cage with females (Friedman test: χ^2^ = 13.32; No. = 10; df = 3; *p* = 0.004); Kendall’s concordance coefficient confirmed that the tendency of the 10 dogs was to rank the four situations in the same order (W = 0.444; *p* = 0.004; alone in cage, alone in cage with daily regular human contact ouside the cage, enriched cage, in cage with two neutered females). In the pairwise comparisons, the Bonferroni correction indicated that the comparison between the situations “alone” and “with females” was significant (adj. sig. *p* = 0.006) (Figure 1).

**Figure 2:**
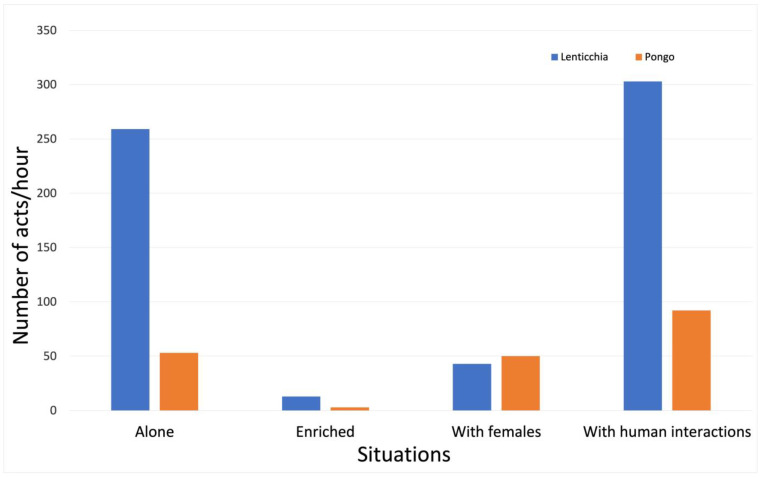
Frequencies of stereotypies by two individuals in the four different situations. The graph shows the frequencies of stereotypic behaviour by Lenticchia and Pongo in the different situations: alone in cage, alone in an enriched cage, in cage with two neutered females, and alone in cage with daily regular human contact ouside the cage.

Only two dogs showed stereotypic behaviour, so there was no point in analysing stereotypies with a statistical test; however, it is interesting to note that the trend was similar to that for displacement activities: Lenticchia’s and Pongo’s frequencies of compulsively licking and chewing objects were higher when the dogs were alone in the cage, even if they had regular daily interactions with human beings, than when they were with females or in the enriched cage (Figure 2).

All other behavioural patterns that were related to daily activities were not statistically significant in the comparisons of the four situations (attention: χ^2^ = 4.48, df = 3, *p* = 0.323; physical activity: χ^2^ = 6.84, df = 3, *p* = 0.077; dozing: χ^2^ = 6.24, df = 3, *p* = 0.1; barking at humans: χ^2^ = 4.61, df = 3, *p* = 0.202; barking at other dogs: χ^2^ = 4.44, df = 3, *p* = 0.218).

Not surprisingly, olfactory investigation of the environment was higher in enriched cages, where dogs had the opportunity to approach several unknown objects that stimulated dogs to sniff (Friedman test: χ^2^ = 13.80, N = 10, df = 3, *p* = 0.003); Kendall’s concordance coefficient confirmed that the tendency of the 10 dogs was to rank the four situations in the same order (enriched cage, in cage with two neutered females, alone in cage, alone in cage with daily regular human contact ouside the cage) (W = 0.460; *p* = 0.003), and the Bonferroni correction for multiple comparisons revealed that the real differences were between the “enriched” and “with human interactions”, and between the “alone” and “enriched” situations (adj. sig. *p* = 0.019 and *p* = 0.034, respectively).

**Figure 3:**
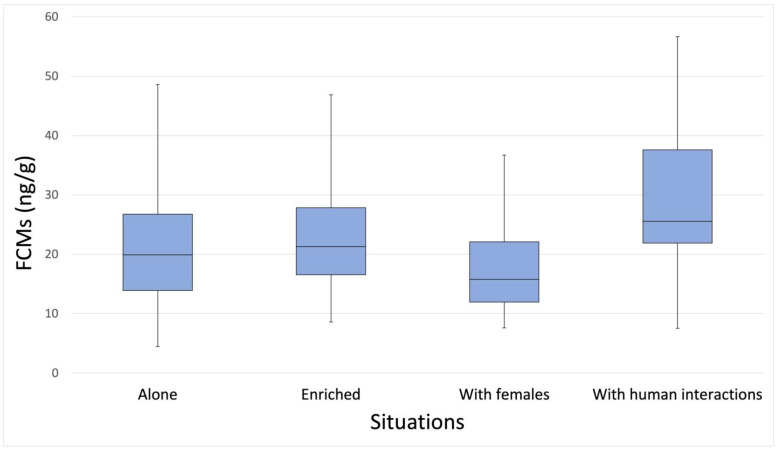
Boxplots of dogs’ average faecal cortisol metabolite (FCM) concentrations in the four different situations. The graph shows the distribution of the average FCM level of each dog in the different situations: alone in cage, alone in an enriched cage, in cage with two neutered females, and alone in cage with regularly human contact. The black bars within the box plots indicate the median; the whiskers represent the min and max value.

**Figure 4:**
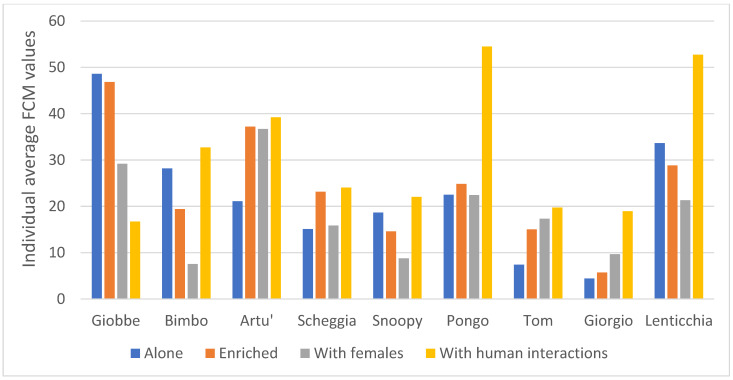
Histogram of individual average faecal cortisol metabolite (FCM) concentrations in the four different situations. The graph shows the average FCM level for each dog in the different situations: alone in cage, alone in an enriched cage, in cage with two neutered females, and alone in cage with regularly human contact.

Finally, average FCM levels of the 10 dogs were highest when the dog was alone in the cage but with regular daily interactions with humans ($\bar{x}$ = 30.2 ng/g), followed by the situation when the dog was in the enriched cage ($\bar{x}$ = 23.7 ng/g; Friedman test: χ^2^ = 8.04, N = 10, df = 3, *p* = 0.045; Figure 3). Again, the lowest average FCM level was found when dogs stayed in cages with females ($\bar{x}$ = 18.3 ng/g) (Kendall’s concordance coefficient: W = 0.268, *p* = 0.045). Post hoc tests underlined that the significant difference was between the “with females” and “with human interactions” situations (*p* = 0.009, Bonferroni adj. sig. *p* = 0.056). As it is common for domestic dogs, individual variability was very high (Figure 4).

## 4. Discussion

Our results suggest that the presence of a conspecific is the best way to reduce stress in shelter dogs: individuals showed a lower frequency of stress related behaviour and also lower FCM levels when other dogs were present. Intraspecific behavioural deprivation can disturb behaviour in dogs [30,31]. Dogs show more unusual behaviours in situations of social isolation [32]. The presence of conspecifics improves environment complexity and positively influences motor and exploratory activity while also reducing the frequency of repetitive and stereotyped behavioural patterns at the same time [19]. Our results support this view; however, other studies [4,5,9,33] found that human contact is also important to reduce stress in sheltered dogs. In particular, Cafazzo et al. [9] provided evidence that a regular walk resulted in a higher total antioxidant capacity in dogs and lowered the frequency of displacement activities and stereotypic behaviour. Coppola et al. [33] suggested that, in fact, human contact may be even more important than contact with another dog. These authors concluded that also environmental enrichment (i.e., toys, beds, food toys and complexity to the enclosure) is important in reducing stress, as well as social interaction (human and conspecific) and adequate exercise.

In this respect, it was not clear if the lowered stress response in dogs studied by Cafazzo et al. [9] was due to the interaction with humans or to the physical exercise, or both. The results of our study seem to lead to the opposite conclusion: the period in which the dogs stayed in the cage alone and had regular daily interactions with a human outside the cage seemed to have been more stressful. We hypothesize that this was probably due to mainly two factors: (i) for dogs that are not accustomed to being taken out of the cage for a walk, it can be stressful to put on a leash and subsequently be led out of the cage; (ii) the route to the outdoor enclosure, where the dog interacted with a human for two hours a day, included passing in front of many cages where dogs inside them barked at dogs that were going to the fenced area. Supporting these hypothesis, Willen et al. and Gunter et al. [34,35] demonstrated that human interaction has limited effects: blood cortisol returned to preinteraction levels within one hour of the dog’s return to the cage. Another hypothesis could be that being exposed to interactions with humans and then be put back in an unenriched cage was stressful for the dogs. Unfortunately, shelters in Italy have strict management protocols so we were limited by what the shelter allowed us to do. This certainly represents an irremediable limitation for this study.

In any case, being alone in the cage, with or without interactions with humans, seemed to be the more stressful condition for the dogs. Stereotyped compulsive behaviours were rare in this sample of dogs; only two dogs, Lenticchia and Pongo, showed a considerably high frequency of stereotypies, which were, again, higher in situations where the dog lived alone in a cage, with and without interaction with humans.

Not surprisingly, dogs showed higher frequencies of sniffing behaviours in the enriched cage. In fact, this situation was set up to provide more visual, olfactory and tactile stimuli for dogs; however, in contrast to the results of other studies (see, e.g., [36]), our results showed a substantial reduction in neither stress-related behaviour nor in FCM levels.

The literature is rich with papers demonstrating that frequent environmental enrichment, frequent intraspecific and/or interspecific interactions, and DAP (dog-appeasing pheromone) or music or adequate exercise decreased stress in shelter-housed dogs (reviewed in [37]). Although even today, no clear consensus exists on how best to measure stress, it is generally agreed that behavioural and physiological parameters should be evaluated [4,5,31,32,35,36]. We decided to utilise behavioural observations and a physiological indicator, i.e., the level of cortisol metabolites in the faeces [24]. Due to the housing conditions of the dogs (most of the time they were housed alone and could not see the human observer), it was not possible to record any affiliative behaviours both towards humans and dogs, so the negative effects of stress were more evident than other aspects of dogs behaviours. Thus, one weak point of this study is the impossibility of collecting data on intra- and interspecific affiliative behaviour, which would have made it possible to assess the positive emotional states of the dogs.

We chose to measure FCMs as a physiological indicator of stress for two main reasons: 1. high level of cortisol has been considered as indicating poor welfare in dogs (reviewed in [37]); 2. measurement of FCMs is less invasive than collecting blood, saliva or urine [38]. This was especially important when studying the shelter dogs, who were not accustomed to being handled. 

Although the individual variability was very high, seven out of ten dogs had a higher value of FCM levels when the dog was alone in the cage, even though it had regular daily interactions with humans. The results suggest then that those situations were the most stressful for our dogs. The lowest FCM levels were recorded when the dog shared the cage with two females; however, displacement activities and FCM levels were not correlated. This is probably due to the well-known tendency for fluctuations in cortisol levels, even if measured in faecal samples, which has made the results of many studies difficult to interpret. Cortisol is an indicator of arousal and is influenced by a number of variables that are impossible to control [39]. Due to management choices, the dogs involved in this study had never gone for a walk outside the cage. Therefore, dogs were not used to the handler who put the collar on the dog, albeit in a gentle manner, and made it go outside to go to the outdoor enclosure. The resulting stress was probably the trigger that increased cortisol levels in the bloodstream and, consequently, in the faeces.

Our study has an added value: it is among the few studies that have assessed the welfare status of dogs not at the time of entry into the shelter but after 1–2 years of permanence, and in different kind of cages. Especially in a country such as Italy, where some dogs remain in a shelter for many years or for their entire life (see, e.g., [40]), it is of crucial importance to assess the adaptation of dogs to long-term stay in a stressful environment. Furthermore, the results suggest that an additional stressor for a dog already adapted to shelter life, may be the change in its daily routine, even if it might seem that the change represents an improvement.

Whatever indicator is utilised, and whatever variable (enrichment, etc.) is used to improve the welfare of dogs, all studies start from a common basis: dogs locked in their cage alone, without environmental enrichment, without intra- and interspecies interactions, without adequate daily exercise, have a poor level of welfare. Therefore, research should take a step forward. This should be, for example, the assessment of how the dog’s quality of life changes over time in a long-term shelter environment; how to monitor these changes; or the assessment of relationships between emotions and behaviour, as suggested by Mellor [41], to give more importance to the affective states of the animals experiencing the permanence in the shelter in an attempt to promote the positive affective states. 

## 5. Conclusions

This study aimed to highlight which variables mostly influence welfare in shelter dogs. According to our results, the presence of conspecifics in the cage seem to be the best solution to reduce stress and anxiety in shelter dogs and improve their life condition. This research could provide some useful guidelines for managing shelters and improving dogs’ life condition.

## Figures and Tables

**Table 1:** The ethogram utilized in the study: the behavioural patterns and relative categories.

Behavioural Categories	Behavioural Patterns
Attention	Raising ears: raising and holding the ears. Looking outside: looking outside the cage. Looking out carefully: looking outside the cage very carefully; the position resembles that described for “prompt”, but the dog is not ready to spring up. Looking at unknown people: looking at people the dog does not know. Looking at volunteer: looking at a shelter volunteer worker. Looking at dog: looking at another dog. Raising foreleg: raising one foreleg. Raising forelegs on wall: raising both forelegs onto the wall or onto the bars, looking carefully outside.
Olfactory investigation	Sniffing the environment: putting the muzzle on the ground, on the wall, or on the objects in the cage. Sniffing air: raising the head, moving the nostrils and breathing the air to perceive odours. Sniffing unknown people: pointing the muzzle towards people the dog does not know; the dog moves the nostrils, clearly trying to perceive their odours. Sniffing volunteer: pointing the muzzle towards volunteers working in the shelter; the dog moves the nostrils, clearly trying to perceive their odours. Sniffing dog: putting the muzzle close or on another dog trying to perceive its odour.
Physical activity	Walking: walking in the cage. Trotting: trotting in the cage. Galloping: galloping in the cage. In/out: going in and out of the indoor/outdoor zone of the cage.
Dozing	Dozing: curling up; the dog is half asleep.
Stereotyped or repetitive behaviour	Repetitive pacing in circles: repetitive walking in a circle within the cage. Licking or biting compulsively: repeatedly licking or biting the bars, the wall and/or objects. Catching flies: trying to catch an imaginary fly with the mouth; clutching at empty air with the teeth. Coprophagy: eating its own or the faeces of other dogs. Self-mutilation: licking itself continuously in same part of the body, so intensely to cause abrasions or even wounds.
Displacement activities	Body shaking: shaking the body quickly sideward. Scratching: raising one hind leg and vigorously scratching part of the body. Muzzle licking: passing the tongue over the muzzle. Yawning: opening the mouth and inhaling and exhaling air. Auto-grooming: cleaning itself with the tongue and the teeth.
Vocalisations	Barking: emitting an abrupt, loud, noisy, and often repetitive vocalisation characteristic of dogs. Whining: emitting a mournful vocalisation. Howling: emitting a vocalisation that consists of a long, high and mournful sound; characteristic of wolves.

**Table 2:** List of the dogs involved in the study and the number of faecal samples collected for each dog in each situation.

Name	Alone in the Cage	Enriched Cage	In Cage with Females	Interactions with Humans
Artù	5	5	5	3
Bimbo	5	6	4	6
Giobbe	8	5	8	7
Giorgio	4	6	5	7
Lenticchia	6	6	5	7
Pongo	4	5	6	6
Puzzola	5	7	7	6
Scheggia	6	6	7	3
Snoopy	4	5	6	4
Tom	3	6	7	5

**Table 3:** Results of correlations between behavioural patterns recorded during moments of silence and moments when all dogs were barking (Spearman rank correlation test). All correlations were significant, with the exception of attention.

Behavioural Categories	N	Rho	*p* (Two Tailed)
Attention	10	0.125	0.443
Sniffing	10	0.387	0.014
Physical activity	10	0.760	0.0001
Dozing	10	0.560	0.0002
Stereotypies	10	0.583	0.0001
Displacement activities	10	0.535	0.0004

## Data Availability

The data presented in this study are available on request from the corresponding author.
